# Experimental exposure assessment for *in vitro* cell-based bioassays in 96- and 384-well plates

**DOI:** 10.3389/ftox.2023.1221625

**Published:** 2023-07-25

**Authors:** Julia Huchthausen, Maria König, Beate I. Escher, Luise Henneberger

**Affiliations:** ^1^ Department of Cell Toxicology, Helmholtz Centre for Environmental Research—UFZ, Leipzig, Germany; ^2^ Department of Geosciences, Environmental Toxicology, Eberhard Karls University Tübingen, Tübingen, Germany

**Keywords:** solid-phase microextraction, bioassay, *in vitro*, high throughput, PPARγ

## Abstract

*In vitro* cell-based bioassays have great potential for applications in the human health risk assessment of chemicals. The quantification of freely dissolved concentrations (*C*_free_) in *in vitro* assays is essential to generate reliable data for *in vitro*-to-*in vivo* extrapolation. Existing methods for the quantification of *C*_free_ are limited to low-throughput microtiter plates. The present study is a proof of principle for the applicability of a solid-phase microextraction (SPME) method for the determination of *C*_free_ in the peroxisome proliferator-activated receptor gamma (PPARγ) bioassay run in 384-well plates with 80 µL medium per well. The effect concentrations obtained from 384-well plates were compared with those obtained from 96-well plates in a previous study. Nominal effect concentrations obtained using 96- and 384-well plates agreed with each other within a factor of three, and freely dissolved effect concentrations agreed within a factor of 6.5. The good degree of agreement in the results from both plate formats proves the general applicability of the SPME method for the determination of *C*_free_ for bioassays in 384-well plates, making the present study a first step toward exposure assessment in high-throughput bioassays.

## 1 Introduction

Chemicals are omnipresent in today’s world. People are exposed to a mix of thousands of different chemicals that could have unknown consequences for human health. For this reason, thorough risk assessment is essential to determine the possible consequences of human exposure to chemicals (Rice et al., 2008). Cell-based bioassays have gained popularity in the field of toxicology in recent years. With the initiation of the “Toxicity Testing in the 21st Century” (Tox21) program in 2007, the US National Research Council paved the way for *in vitro* bioassays as alternatives to animal testing (US National Research Council, 2007).

Cell-based bioassays allow for automatization, making them a cost-effective and time-saving method for the assessment of effects. However, chemical exposure during the assay must be monitored in order to obtain biologically relevant data (Gülden et al., 2002; Henneberger et al., 2019b; Huchthausen et al., 2020). Complementary to the typically used nominal concentration (*C*_nom_), freely dissolved concentration (*C*_free_) in the assay medium should be used as a concentration metric if chemical losses, e.g., due to sorption to medium components, cannot be excluded (Groothuis et al., 2015).

Various mass balance models are available to predict *C*_free_ (Armitage et al., 2021; Proença et al., 2021). However, previous studies from our group have shown that, especially for ionizable chemicals, model predictions can deviate widely from experimental values (Henneberger et al., 2020; Huchthausen et al., 2020). Measuring *C*_free_ during the assay at effective concentrations has been demonstrated to be especially important for ionizable chemicals that show non-linear binding to medium proteins (Henneberger et al., 2019a; Henneberger et al., 2019b; Huchthausen et al., 2020).

We have previously developed a method integrating exposure assessment of single chemicals with diverse physicochemical properties and chemical mixtures into a routine bioassay workflow in 96-well plates (Henneberger et al., 2019b; Huchthausen et al., 2020; Henneberger et al., 2022). Solid-phase microextraction (SPME) was established by Heringa et al. (2003) to be a suitable method for experimental exposure assessment and has been shown to be a good method for measurement of *C*_free_ in bioassay medium aliquots from 96-well plates (Henneberger et al., 2019b; Huchthausen et al., 2020). Cell-based bioassays are often performed in automated high-throughput screening (HTS) systems using 384- or 1,536-well plates (Inglese and Auld, 2008). High-tier well plate formats allow higher sample throughput without a linear increase in costs (Kolb and Neumann, 1997); however, they are more challenging with regard to exposure assessment due to the low sample volume (of typically 40–80 μL for 384-well plates and 6–10 µL for 1,536-well plates), the higher surface area, and the large number of samples per plate.

The aim of this work was to prove that the existing method for the determination of *C*_free_ for 96-well plates can be adapted to bioassay medium aliquots from 384-well plates. The assay used for optimization was the peroxisome proliferator-activated receptor gamma (PPARγ) assay representing the chemical activation of human lipid metabolism. Nine previously tested PPARγ activators were selected, for which *C*_free_ had already been determined experimentally in 96-well plates (Huchthausen et al., 2020). The intent of this study was to demonstrate the general comparability of the measured *C*_free_ values obtained from both plate formats.

## 2 Materials and methods

### 2.1 Solvents and chemicals

To compare the effect concentrations obtained from bioassays conducted in 96- and 384-well plates, nine chemicals previously identified as PPARγ activators (Henneberger et al., 2019b; Huchthausen et al., 2020) were tested: caffeine (neutral), lamotrigine (base), six acids (diclofenac, 2,4-dichlorophenoxyacetic acid (2,4-D), naproxen, ibuprofen, torasemide, and warfarin), and telmisartan (multifunctional). The chemical class, logarithmic liposome–water distribution ratio (log *D*_lip/w_), acidity constant (p*K*_a_), and speciation at pH 7.4 of each of the test chemicals are shown in Table 1. Information on the measurement of p*K*_a_ is provided in Supplementary Data Sheet 1 (Text S1). Further information on the chemicals can be found in Supplementary Data Sheet 1 (Table S1).

**TABLE 1:** Test chemicals, their logarithmic liposome–water distribution ratio (log *D*_lip/w_), their chemical class, p*K*_a_, and speciation at pH 7.4.

Chemical	Log *D*_lip/w_ (L_w_/L_lip_)	Chemical class	Acidity constant (p*K*_a_)	Speciation at pH 7.4
Caffeine	0.08^a^	Neutral	-	100% neutral
Lamotrigine	2.06^b^	Base	5.53^c^	99% neutral and 1% cationic
Diclofenac	2.64^d^	Acid	4.07^e^	>99% anionic
2,4-D	2.02^d^	Acid	2.70^c^	>99% anionic
Naproxen	2.17^d^	Acid	4.19^c^	>99% anionic
Ibuprofen	1.81^f^	Acid	4.37^c^	>99% anionic
Torasemide	2.05^d^	Acid	6.77^c^	81% anionic and 19% neutral
Warfarin	1.62^d^	Acid	4.95^c^	>99% anionic
Telmisartan	4.73^g^	Multifunctional	3.07, 4.34, and 6.04^e^	96% anionic and 4% zwitterionic

^a^ Predicted using the UFZ-LSER database (Ulrich et al., 2017).

^b^ Henneberger et al. (2020).

^c^Measured using Sirius T3 (this study).

^d^ Henneberger et al. (2019a).

^e^ Niu et al. (2022).

^f^ Avdeef et al. (1998).

^g^ Huchthausen et al. (2020).

LC-MS-grade acetonitrile, isopropanol (CHEMSOLUTE), methanol, and formic acid (Honeywell) with ≥99.9% purity were used. Deionized water was obtained using a Milli-Q water purification system (Merck).

### 2.2 *In vitro* bioassay

The GeneBLAzer PPARγ-UAS-bla HEK 293H cell line was purchased from Thermo Fisher Scientific (catalog number K1419). Cells were seeded at 6,500 cells per well in 30 μL PPARγ assay medium (98% Opti-MEM, 2% charcoal-stripped fetal bovine serum (FBS), 100 U/mL penicillin–streptomycin, and 10 mM HEPES) in BioCoat™ TC-treated 384-well microplates with a poly-D-lysine coating from Corning (catalog number 356663) and incubated at 37°C, 5% CO_2_, and 100% humidity for 24 h. All chemicals were tested in duplicate in linear dilution in the PPARγ assay. In a glass-coated 96-well plate (Thermo Fisher Scientific, catalog number 60180-P300), 150 µL PPARγ assay medium per well was spiked with different chemical concentrations using a digital dispenser (D300e, Tecan). Subsequently, 50 μL spiked assay medium was transferred manually in duplicate from each well of the 96-well dosing plate to the 384-well cell plate, leading to a total volume of 80 µL per well. After dosing, the cells were incubated at 37°C, 5% CO_2_, and 100% humidity for 24 h. The confluency of the cells was measured before and 24 h after dosing using an Incucyte S3 Automated Live-Cell Imager (Essen BioScience) to determine cell viability. PPARγ activation was detected using the LiveBLAzer™ FRET-B/G Loading Kit from Thermo Fisher Scientific (catalog number K1095). The protocol for the PPARγ assay is described in detail in Neale et al. (2017). Medium aliquots of 30 µL from the highest concentration in the dosing plate (t = 0 h) and each well of the cell plate after 24-h incubation (t = 24 h) were transferred to amber glass vials with inserts from LABSOLUTE (catalog number 7648146) for the determination of *C*_free_.

### 2.3 Determination of *C*_free_

Custom-made metal alloy SPME fibers from Sigma-Aldrich coated with C18/polyacrylonitrile (approximately 30 mm total length, 2 mm coating length, 45 µm coating thickness, and approximately 69 nL coating volume), were used.

The fibers were conditioned in methanol for 24 h and in Milli-Q water for 20 min. They were immersed into the medium aliquots (30 µL) and incubated at 37°C and 1,800 rpm (BioShake iQ, QInstruments) for 24 h. The fibers were moved to a new vial containing 60 µL of the respective desorption solvent (Supplementary Table S1) and desorbed at 37°C and 1,800 rpm for 2 h. The protocol for the SPME method is described in detail in Henneberger et al. (2019a). Controls in phosphate-buffered saline (PBS) were run separately for each chemical to determine the fiber–water distribution ratio (*D*_f/w_).

The chemical concentration in the PBS controls and the desorption solution of all samples was determined using an Agilent 1260 Infinity II liquid chromatograph coupled to an Agilent 6420 Triple Quad mass spectrometer equipped with a Luna Omega Polar C18 column, a BioZen peptide PS-C18 column, or a Kinetex C18 column. Further details on the instrumentation can be found in Supplementary Data Sheet 1 (Tables S2 and S3). Acetonitrile blanks and standard solutions in the respective solvent (1–10,000 ng/mL) were measured with the samples.

### 2.4 Data evaluation

To obtain concentration response curves (CRCs) from the bioassay results, *C*_nom_ or *C*_free_ values were plotted against the measured effects. The nominal or freely dissolved concentration leading to a cytotoxicity of 10% is the IC_10_ or IC_10,free_ value; this was calculated from the slope of the linear part of the CRC using Eq. 1. The standard error of the IC_10_ or IC_10,free_ was calculated using Eq. 2 (Escher et al., 2018).

$${IC}_{10,\mathrm{free}}=\frac{10\%}{slope},$$ (1)

$$SE\;\left({IC}_{10,\mathrm{free}}\right)=\frac{10\%}{{slope}^{2}}\times SE\;\left(slope\right).$$ (2)

The reference chemical for the PPARγ assay was rosiglitazone. This was dosed in duplicate on each plate in addition to the test chemicals to determine the maximum effect. The nominal or freely dissolved concentration leading to 10% of the maximum effect is referred to as the EC_10_ or EC_10,free_, respectively. This value was calculated from the linear part of the CRC at concentrations below IC_10_ or IC_10,free_ using Eq. 3. The standard error of the EC_10_ was calculated using Eq. 4.

$${IC}_{10,\mathrm{free}}=\frac{10\%}{slope},$$ (3)

$$SE\;\left({IC}_{10,\mathrm{free}}\right)=\frac{10\%}{{slope}^{2}}\times SE\;\left(slope\right).$$ (4)

The concentration of the test chemical in the SPME fiber (*C*_f_) was calculated using Eq. 5. *C*_des_ represents the concentration in the desorption solution, *V*_des_ represents the volume of the desorption solution, and *V*_f_ represents the volume of the fiber coating.

$$C_{f}=\frac{C_{des}\times V_{des}}{V_{f}}.$$ (5)

The *D*_f/w_ value was determined from PBS controls using Eq. 6. *C*_w_ represents the chemical concentration in the PBS phase after SPME.

Dfw=CfCw. (6)

*C*_free_ in the medium aliquots was calculated using Eq. 7 (Henneberger et al., 2019b). The total amount of the chemical (*n*_total_) was calculated from *C*_nom_.

Cfree=ntotalDfw×ntotalCf−Vf. (7)

## 3 Results and discussion

The measured effects (cytotoxicity and PPARγ activation) were plotted against *C*_nom_ and *C*_free_. CRCs for all chemicals are shown in Supplementary Data Sheet 1 (Figure S1).

Seven of the nine test chemicals were cytotoxic (all except naproxen and torasemide), and all activated PPARγ. Comparisons of the nominal IC_10_ and EC_10_ to the measured IC_10,free_ and EC_10,free_ are presented in Table 2.

**TABLE 2:** Nominal and freely dissolved effect concentrations of the test chemicals as measured in experiments conducted in 384-well plates. IC_10,nom_ or IC_10,free_: Nominal or freely dissolved concentration leading to a cytotoxicity of 10%. EC_10,nom_ or EC_10,free_: Nominal concentration leading to 10% of the maximum effect. CV: Coefficient of variation.

	Cytotoxicity				PPARγ activation			
**Chemical**	**IC**_**10,nom**_ **[M]**	**CV [%]**	**IC**_**10,free**_ **[M]**	**CV [%]**	**EC**_**10,nom**_ **[M]**	**CV [%]**	**EC**_**10,free**_ **[M]**	**CV [%]**
Caffeine	2.14 × 10^−3^	14.7	1.89 × 10^−3^	14.6	2.49 × 10^−4^	8.4	2.06 × 10^−4^	9.0
Lamotrigine	5.75 × 10^−4^	23.7	4.66 × 10^−4^	22.6	2.68 × 10^−4^	15.9	2.14 × 10^−4^	15.9
Diclofenac	4.99 × 10^−5^	21.9	3.41 × 10^−5^	22.0	2.07 × 10^−6^	42.0	4.84 × 10^−7^	37.5
2,4-D	1.60 × 10^−4^	28.1	1.04 × 10^−4^	28.1	1.31 × 10^−4^	21.7	9.95 × 10^−5^	20.5
Naproxen	Not active up to 3.13 × 10^−4^ M		Not active up to 1.48 × 10^−4^ M		2.15 × 10^−5^	15.8	7.11 × 10^−7^	19.5
Ibuprofen	3.41 × 10^−4^	24.5	1.22 × 10^−4^	22.1	8.90 × 10^−6^	14.8	2.11 × 10^−6^	14.9
Torasemide	Not active up to 3.75 × 10^−4^ M		Not active up to 7.53 × 10^−5^ M		1.70 × 10^−5^	11.6	2.80 × 10^−6^	11.5
Warfarin	2.17 × 10^−4^	17.1	1.15 × 10^−4^	14.9	6.99 × 10^−6^	11.3	4.30 × 10^−7^	16.0
Telmisartan	1.84 × 10^−5^	6.3	1.74 × 10^−6^	7.8	2.96 × 10^−7^	11.7	2.36 × 10^−8^	16.5

For neutral hydrophilic chemicals (lamotrigine and caffeine), the difference between the nominal and freely dissolved effect concentrations determined was less than a factor of 1.3, as these do not bind strongly to proteins or lipids of the assay medium (Henneberger et al., 2019b). For most of the acidic chemicals (diclofenac, naproxen, warfarin, and torasemide), the deviation between the IC_10_ and IC_10,free_ was also less than a factor of 2, but for the EC_10_ and EC_10,free_, the deviation was much higher (up to a factor of 30). This difference is caused by the non-linear binding of acidic chemicals to medium proteins, resulting in higher binding affinity and consequently a lower free fraction of the chemical at low concentrations (Henneberger et al., 2019a; Qin et al., 2023).

To test the applicability of the developed SPME method for the determination of freely dissolved effect concentrations for bioassays run in 384-well plates, the effect concentrations determined in the present study (freely dissolved and nominal) were compared with those from a previous study using 96-well plates (Huchthausen et al., 2020) (Table 3).

**TABLE 3:** Nominal and freely dissolved effect concentrations of the test chemicals as measured in experiments conducted in 96-well plates plates [Adapted with permission from Huchthausen et al. (2020). Copyright (2020) American Chemical Society]. IC_10,nom_ or IC_10,free_: Nominal or freely dissolved concentration leading to a cytotoxicity of 10%. EC_10,nom_ or EC_10,free_: Nominal concentration leading to 10% of the maximum effect. CV: Coefficient of variation.

	Cytotoxicity				PPARγ activation			
**Chemical**	**IC**_**10,nom**_ **[M]**	**CV [%]**	**IC**_**10,free**_ **[M]**	**CV [%]**	**EC**_**10,nom**_ **[M]**	**CV [%]**	**EC**_**10,free**_ **[M]**	**CV [%]**
Caffeine	2.44 × 10^−3^	18.2	1.84 × 10^−3^	13.7	3.48 × 10^−4^	6.6	2.89 × 10^−4^	8.9
Lamotrigine	4.39 × 10^−4^	13.8	3.82 × 10^−4^	15.6	1.02 × 10^−4^	10.5	9.81 × 10^−5^	10.7
Diclofenac	Cytotoxicity oberced, but <10%				2.12 × 10^−6^	11.0	2.75 × 10^−7^	10.4
2,4-D	Not active up to 4.64 × 10^−4^ M		Not active up to 2.19 × 10^−4^ M		4.92 × 10^−5^	3.1	2.28 × 10^−5^	4.3
Naproxen	Not active up to 3.86 × 10^−4^ M		Not active up to 3.27 × 10^−4^ M		1.77 × 10^−5^	4.2	4.62 × 10^−6^	5.3
Ibuprofen	3.16 × 10^−4^	15.7	1.50 × 10^−4^	11.1	5.46 × 10^−6^	9.1	8.78 × 10^−7^	11.1
Torasemide	1.84 × 10^−4^	7.7	7.19 × 10^−6^	6.7	5.19 × 10^−5^	8.1	2.12 × 10^−6^	8.3
Warfarin	2.42 × 10^−4^	10.5	1.04 × 10^−4^	15.2	4.01 × 10^−6^	5.2	3.67 × 10^−7^	10.6
Telmisartan	2.37 × 10^−5^	12.4	6.32 × 10^−6^	12.5	1.67 × 10^−7^	9.6	2.58 × 10^−8^	33.4

**FIGURE 1:**
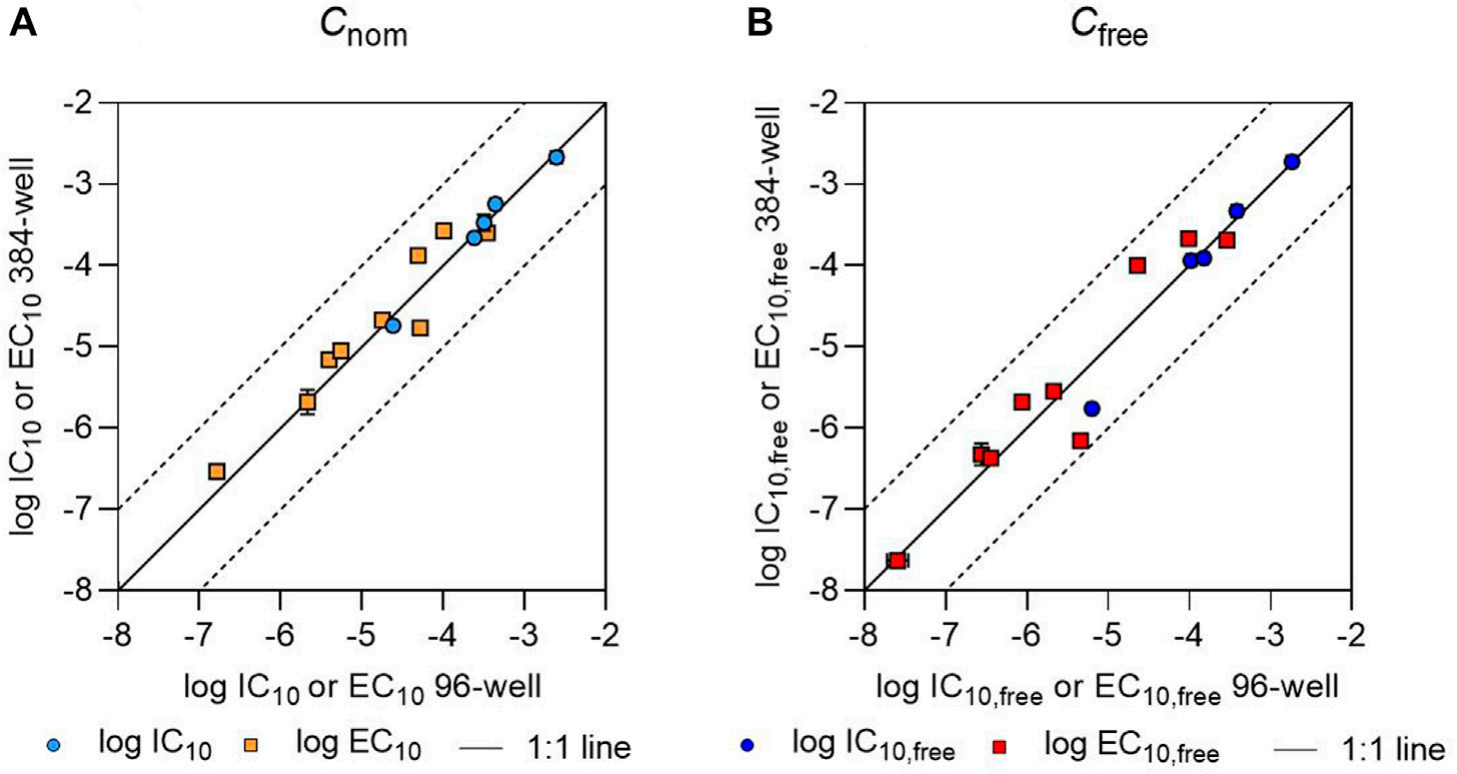
Comparison of **(A)** logarithmic nominal effect concentrations (log IC_10_ or log EC_10_) and **(B)** logarithmic freely dissolved effect concentrations (log IC_10,free_ or log EC_10,free_) as determined in 96- and 384-well plates. The IC_10_ and EC_10_ values and IC_10,free_ and EC_10,free_ values for 96-well plates have been previously published (Huchthausen et al., 2020). The black solid line indicates a 1:1 correlation between the values; the black dashed lines indicate deviations from this by a factor of 10. Error bars indicate the standard deviation of the effect concentration values, but are often smaller than the size of the data points.

All IC_10_ values determined in 384-well plates differed by less than a factor of 2 from the IC_10_ values determined in 96-well plates. However, not all chemicals showed cytotoxicity in both plate formats. Naproxen was non-cytotoxic up to a concentration of 3.86 × 10^−4^ M in both plate types; torasemide had an IC_10_ value of 1.84 × 10^−4^ M in 96-well plates but did not show cytotoxicity up to 3.75 × 10^−4^ M in 384-well plates; and no IC_10_ value could be measured for 2,4-D or diclofenac in 96-well plates, but their IC_10_ values were 1.60 × 10^−4^ M and 4.99 × 10^−5^ M, respectively, in 384-well plates. The EC_10_ values measured in 384- and 96-well plates also deviated from one another by less than a factor of 2, with the exception of torasemide, for which the EC_10_ value deviated by a factor of 3 (Figure 1).

The IC_10,free_ and EC_10,free_ values determined in the present study were also consistent with our previous results from 96-well plates (Huchthausen et al., 2020). The IC_10,free_ values for warfarin, ibuprofen, lamotrigine, and caffeine determined based on 30 µL aliquots from 384-well plates differed by less than a factor of 2 from the concentrations determined based on 180 µL aliquots from 96-well plates (Huchthausen et al., 2020). For telmisartan, the ratio between the determined IC_10,free_ values was slightly higher, with deviation by a factor of 4. EC_10,free_ values determined in each of the two well plate formats were also within a factor of 2 for most of the chemicals, with the exceptions of 2,4-D (whose values deviated by a factor of 4.4) and naproxen, which showed the highest deviation with a factor of 6.5. The higher deviations seen in the case of EC_10,free_ values compared to IC_10,free_ values could have been caused by higher measurement errors in the LC-MS/MS analysis at low concentrations, since the EC_10,free_ values were often orders of magnitude lower than the IC_10,free_ values. Specifically, naproxen is highly bound to proteins at low concentrations, leading to freely dissolved concentrations close to the limit of quantification (Henneberger et al., 2019a).

Comparing the relative standard deviations of the effect concentrations, it is noticeable that the coefficient of variation was generally higher in the case of 384-well plate measurements for both nominal and freely dissolved effect concentrations (Table 2; Table 3). This may have been caused by experimental inaccuracies due to the lower sample volume.

Additionally, an unpaired *t*-test was performed for a more detailed comparison of nominal and freely dissolved effect concentrations obtained using both plate formats (Supplementary Data Sheet 1: Figure S2; Supplementary Table S4). This *t*-test indicated that there was no significant difference between the plate formats (*p*-value >0.05) for the majority of effect concentrations, both nominal and freely dissolved. There was a significant difference between the effect concentrations measured in 96- and 384-well plates for torasemide and warfarin (nominal concentration); diclofenac, naproxen, and ibuprofen (freely dissolved concentration); and lamotrigine and telmisartan (both measures). However, the difference between the two plate formats was not highly significant for most of the chemicals and effect concentrations, with the exception of telmisartan. These differences might therefore have been caused by variations in the bioassay setup and by biological variability.

## 4 Conclusion

Measurement of freely dissolved effect concentrations is necessary for quantitative *in vitro*-to-*in vivo* extrapolation (QIVIVE) and to increase the relevance of *in vitro* tests for risk assessment (Villeneuve et al., 2019). For several test chemicals examined in the present study and in our previous publication, measurement of *C*_free_ at all dosed concentrations was found to be the best method of deriving the freely dissolved effect concentration (Huchthausen et al., 2020), given that non-linear sorption isotherms are only available for a small number of chemicals and currently available *in vitro* exposure models are not applicable if the chemical concentration decreases over time.

The results of the present study show that the previously developed SPME method can also be applied to sample volumes as small as 30 µL from 384-well plates without changing the assay workflow or reducing sample throughput. Through the use of 384-well plates, sample throughput can be quadrupled compared to 96-well plates, making these results the first step in the development of a high-throughput SPME method for the determination of *C*_free_ in bioassay medium aliquots. The advantages of the SPME method over more conventional methods, such as rapid equilibrium dialysis (RED), are the plastic-free system and the use of samples without dilution. The next step will be to automate the SPME method, because the high number of samples resulting from the use of 384-well plates cannot easily be handled manually. Automation could significantly speed up concentration measurement, so that the throughput of the chemical analysis would become similar to the throughput of the bioassays. We recently developed an automated SPME workflow using a Supel™ BioSPME 96-Pin Device (Supelco); however, the method is still limited to comparably high sample volumes (600 µL) in 96 deep-well plates (Huchthausen et al., 2022).

In summary, we have demonstrated that the previously published method for concentration measurements in 96-well plates is applicable for high-throughput assays conducted in 384-well plates without modification of the assay procedure. *C*_free_ can be determined from small medium aliquots as low as 30 µL in parallel to the assay to provide nominal effect concentrations and freely dissolved effect concentrations for QIVIVE. The validation conducted in this proof-of-principle study was only performed in one bioassay with a small number of chemicals. For a complete validation, the comparison of a wide range of chemicals with diverse physicochemical properties and in different assays using media with different FBS contents is necessary. However, by establishing and automating this method, exposure assessment can be simplified, and more meaningful toxicity data can be generated on a routine basis.

## Data Availability

The original contributions presented in the study are included in the article/Supplementary Material; further inquiries can be directed to the corresponding author.

## References

[B1] Armitage J. M. Sangion A. Parmar R. Looky A. B. Arnot J. A. (2021). Update and evaluation of a high-throughput *in vitro* mass balance distribution model: IV-MBM EQP v2.0. Toxics 9 (11), 315. 10.3390/toxics9110315 34822706 PMC8625852

[B2] Avdeef A. Box K. J. Comer J. E. A. Hibbert C. Tam K. Y. (1998). pH-metric logP 10 Determination of liposomal membrane-water partition coefficients of ionizable drugs. Pharm. Res. 15 (2), 209–215. 10.1023/a:1011954332221 9523305

[B3] Escher B. I. Neale P. A. Villeneuve D. L. (2018). The advantages of linear concentration-response curves for *in vitro* bioassays with environmental samples. Environ. Toxicol. Chem. 37 (9), 2273–2280. 10.1002/etc.4178 29846006 PMC6150494

[B4] Groothuis F. A. Heringa M. B. Nicol B. Hermens J. L. M. Blaauboer B. J. Kramer N. I. (2015). Dose metric considerations in *in vitro* assays to improve quantitative *in vitro*-*in vivo* dose extrapolations. Toxicology 332, 30–40. 10.1016/j.tox.2013.08.012 23978460

[B5] Gülden M. Morchel S. Tahan S. Seibert H. (2002). Impact of protein binding on the availability and cytotoxic potency of organochlorine pesticides and chlorophenols *in vitro* . Toxicology 175 (1-3), 201–213. 10.1016/s0300-483x(02)00085-9 12049848

[B6] Henneberger L. Huchthausen J. König M. Menge A. Wojtysiak N. Escher B. I. (2022). Experimental exposure assessment of designed chemical mixtures in cell-based *in vitro* bioassays. Front. Environ. Chem. 3, 1018162. 10.3389/fenvc.2022.1018162

[B7] Henneberger L. Mühlenbrink M. Fischer F. C. Escher B. I. (2019a). C18-coated solid-phase microextraction fibers for the quantification of partitioning of organic acids to proteins, lipids, and cells. Chem. Res. Toxicol. 32 (1), 168–178. 10.1021/acs.chemrestox.8b00249 30585484

[B8] Henneberger L. Mühlenbrink M. Heinrich D. J. Teixeira A. Nicol B. Escher B. I. (2020). Experimental validation of mass balance models for *in vitro* cell-based bioassays. Environ. Sci. Technol. 54 (2), 1120–1127. 10.1021/acs.est.9b06144 31852189

[B9] Henneberger L. Mühlenbrink M. König M. Schlichting R. Fischer F. C. Escher B. I. (2019b). Quantification of freely dissolved effect concentrations in *in vitro* cell-based bioassays. Archives Toxicol. 93 (8), 2295–2305. 10.1007/s00204-019-02498-3 31230094

[B10] Heringa M. B. Schreurs R. van der Saag P. T. van der Burg B. Hermens J. L. M. (2003). Measurement of free concentration as a more intrinsic dose parameter in an *in vitro* assay for estrogenic activity. Chem. Res. Toxicol. 16 (12), 1662–1663.

[B11] Huchthausen J. Henneberger L. Mälzer S. Nicol B. Sparham C. Escher B. I. (2022). High-throughput assessment of the abiotic stability of test chemicals in *in vitro* bioassays. Chem. Res. Toxicol. 35 (5), 867–879. 10.1021/acs.chemrestox.2c00030 35394761

[B12] Huchthausen J. Mühlenbrink M. König M. Escher B. I. Henneberger L. (2020). Experimental exposure assessment of ionizable organic chemicals in *in vitro* cell-based bioassays. Chem. Res. Toxicol. 33 (7), 1845–1854. 10.1021/acs.chemrestox.0c00067 32368900

[B13] Inglese J. Auld D. S. (2008). “High throughput screening (HTS) techniques: Applications in chemical biology,” in Wiley encyclopedia of chemical biology. Editor Begley T. P., 1–15. 10.1002/9780470048672.wecb223

[B14] Kolb A. J. Neumann K. (1997). Beyond the 96-well microplate: Instruments and assay methods for the 384-well format. J. Biomol. Screen. 2 (2), 103–109. 10.1177/108705719700200209

[B15] Neale P. A. Altenburger R. Ait-Aissa S. Brion F. Busch W. de Aragao Umbuzeiro G. (2017). Development of a bioanalytical test battery for water quality monitoring: Fingerprinting identified micropollutants and their contribution to effects in surface water. Water Res. 123, 734–750. 10.1016/j.watres.2017.07.016 28728110

[B16] Niu L. Henneberger L. Huchthausen J. Krauss M. Ogefere A. Escher B. I. (2022). pH-dependent partitioning of ionizable organic chemicals between the silicone polymer polydimethylsiloxane (PDMS) and water. ACS Environ. Au 2 (3), 253–262. 10.1021/acsenvironau.1c00056 37102138 PMC10114720

[B17] Proença S. Escher B. Fischer F. Fisher C. Grégoire S. Hewitt N. (2021). Effective exposure of chemicals in *in vitro* cell systems: A review of chemical distribution models. Toxicol. vitro 73, 105133. 10.1016/j.tiv.2021.105133 33662518

[B18] Qin W. Henneberger L. Huchthausen J. König M. Escher B. I. (2023). Role of bioavailability and protein binding of four anionic perfluoroalkyl substances in cell-based bioassays for quantitative *in vitro* to *in vivo* extrapolations. Environ. Int. 173, 107857. 10.1016/j.envint.2023.107857 36881956

[B19] Rice G. MacDonell M. Hertzberg R. C. Teuschler L. Picel K. Butler J. (2008). An approach for assessing human exposures to chemical mixtures in the environment. Toxicol. Appl. Pharmacol. 233 (1), 126–136. 10.1016/j.taap.2008.05.004 18589469

[B20] Ulrich N. Endo S. Brown T. N. Watanabe N. Bronner G. Abraham M. H. (2017). UFZ-LSER database v 3.2.1. Available at: http://www.ufz.de/lserd (Accessed July 07, 2023).

[B21] US National Research Council (2007). Toxicity testing in the 21st century. Washington, DC: The National Academies Press.

[B22] Villeneuve D. L. Coady K. Escher B. I. Mihaich E. Murphy C. A. Schlekat T. (2019). High-throughput screening and environmental risk assessment: State of the science and emerging applications. Environ. Toxicol. Chem. 38 (1), 12–26. 10.1002/etc.4315 30570782 PMC6698360

