# Supplementary material for: Experimental exposure assessment for in vitro cell-based bioassays in 96- and 384-well plates
Source: Front Toxicol. 2023 Jul 25;5:1221625. doi: 10.3389/ftox.2023.1221625 (PMC10411540; doi:10.3389/ftox.2023.1221625)
Supplement: Supplementary file 1 [file DataSheet1.PDF]

*Supplementary Material*

# Experimental exposure assessment for *in vitro* cell-based bioassays in 96- and 384-well plates

**Julia Huchthausen,**[†] **Maria König,**[†] **Beate I. Escher,**[†,‡] **Luise Henneberger**[†,*]

[†] Helmholtz Centre for Environmental Research – UFZ, Department of Cell Toxicology, Permoserstr. 15, 04318 Leipzig, Germany

[‡] Eberhard Karls University Tübingen, Environmental Toxicology, Department of Geosciences, 72076 Tübingen, Germany

***Correspondence:**
Luise Henneberger,
luise.henneberger@ufz.de.

## S1: Test chemicals

**Text S1:** p$K_a$ measurement.

The acidity constants (p$K_a$) of six of the test chemicals were measured using a Sirius T3 titrator (Pion). A detailed description of the experimental procedure can be found in the literature (Niu et al, 2022). Briefly, 5 µL of a 10 mM DMSO stock solution of each test chemical and 25 µL of a phosphate buffer (14.4 mM $K_2HPO_4$ and 0.15 M KCl) were added to a Sirius T3 test vial. A reference vial containing 5 µL DMSO (Roth, A994-100 ML) and 25 µL phosphate buffer was measured with each sample. The p$K_a$ measurement was performed using the automated UV-metric p$K_a$ protocol of the Sirius T3 Control software (version 2.0.0.0.). Three sequential titrations were performed with the same sample and a constant ionic strength of 0.15 M KCl ranging from pH 2 to 12 by adding 0.5 M HCl or 0.5 M KOH while UV absorbance was measured. Data analysis was performed using Sirius T3 Refine software (version 2.0.0.0.). All p$K_a$ values were reported as the mean of the three titrations (or six for 2,4-D). The p$K_a$ of ibuprofen was measured in the presence of 25 – 52 % methanol with 0.15 M KCl. The measured p$K_a$ was extrapolated to the p$K_a$ at 0 % methanol with Yasuda-Shedlovsky extrapolation (Shedlovsky, 1962; Yasuda, 1959).

**Table S1:** Test chemicals, their chemical class, CAS number, purity, supplier and SPME desorption solution.

| ***Chemical*** | ***Chemical class*** | ***CAS*** | ***Purity*** | ***Supplier*** | ***SPME desorption solution*** |
|---|---|---|---|---|---|
| caffeine | neutral | 58-08-2 | ≥99% | Sigma-Aldrich | 10/90 ACN/$H_2O$ |
| lamotrigine | base | 84057-84-1 | ≥98% | Cayman Chemical Company | 50/50 ACN/$H_2O$ |
| diclofenac sodium | acid | 15307-79-6 | ≥99% | Cayman Chemical Company | 90/10 ACN/$H_2O$ |
| 2,4-dichloro-phenoxyacetic acid (2,4-D) | acid | 94-75-7 | ≥97% | Cayman Chemical Company | 50/50 MeOH/$H_2O$ |
| (*S*)-naproxen | acid | 22204-53-1 | ≥99% | Cayman Chemical Company | 50/50 MeOH/$H_2O$ |
| ibuprofen | acid | 15687-27-1 | ≥99.6% | Euro OTC Pharma | 50/50 MeOH/$H_2O$ |
| torasemide | acid | 56211-40-6 | ≥98% | Sigma-Aldrich | 50/50 MeOH/$H_2O$ |
| warfarin | acid | 81-81-2 | ≥99% | Sigma-Aldrich | 50/50 MeOH/$H_2O$ |
| telmisartan | multifunctional | 144701-48-4 | ≥98% | Cayman Chemical Company | 90/10 ACN/$H_2O$ |

## S2: Instrumental analysis

**Table S2:** LC parameters for the test chemicals used in this study. The eluents used are composed as follows: A) 5 % acetonitrile and 95 % water, B) 100 % acetonitrile, C) 5 % acetonitrile and 95 % water with 0.1 % formic acid, D) 95 % acetonitrile and 5 % water with 0.1 % formic acid.

| *Chemical* | *Column* | *Eluent* | *Retention time [min]* | *Flow rate [mL/min]* | *Injection volume [µL]* |
|---|---|---|---|---|---|
| caffeine | Phenomenex Luna Omega Polar C18 1.6 µm 50 × 2.1 mm, 25°C | 95 % A 5 % B | 1.75 | 0.5 | 1 |
| lamotrigine | BioZen peptide PS-C18 1.6 µm 50 × 2.1 mm, 25°C | 90 % C 10 % D | 0.79 | 0.5 | 1 |
| diclofenac | Phenomenex Kinetex C18 1.7 µm 50 × 2.1 mm, 30°C | 50 % C 50 % D | 1.27 | 0.5 | 1 |
| 2,4-D | Phenomenex Kinetex C18 1.7 µm 50 × 2.1 mm, 40°C | 60 % C 40 % D | 0.96 | 0.5 | 1 |
| naproxen | Phenomenex Kinetex C18 1.7 µm 50 × 2.1 mm, 40°C | 55 % C 45 % D | 0.83 | 0.5 | PBS: 2 Desorption solution: 15 |
| ibuprofen | Phenomenex Kinetex C18 1.7 µm 50 × 2.1 mm, 40°C | 45 % C 55 % D | 0.98 | 0.5 | 5 |
| torasemide | Phenomenex Kinetex C18 1.7 µm 50 × 2.1 mm, 40°C | 75 % C 25 % D | 0.95 | 0.5 | 1 |
| warfarin | Phenomenex Kinetex C18 1.7 µm 50 × 2.1 mm, 30°C | 55 % C 45 % D | 1.2 | 0.5 | 1 |
| telmisartan | Phenomenex Kinetex C18 1.7 µm 50 × 2.1 mm, 40°C | 90 % C 10 % D | 0.82 | 0.5 | 1 |

**Table S3:** MS parameters for the test chemicals used in this study.

| *Chemical* | *Ion source* | *Frag-mentor voltage [V]* | *MRM transitions (Collision energy [V])* | *Source parameters: capillary voltage [V]; gas flow [ml/min]; gas temperature [°C]; nebulizer [psi]* |
|---|---|---|---|---|
| caffeine | ESI+ | 110 | 195.2 → 138/110.1 (17/25) | 5000; 13; 310; 60 |
| lamotrigine | ESI+ | 150 | 256 → 211/145 (26/45) | 3000; 13; 290; 50 |
| diclofenac | ESI- | 80 | 294 → 249.8/213.9 (9/21) | 2000; 13; 350; 25 |
| 2,4-D | ESI- | 80 | 218.96 → 160.9/124.9 (12/28) | 2000; 13; 350; 25 |
| naproxen | ESI- | 80 | 229.08 → 185/168.9 (1/33) | 5000; 13; 290; 25 |
| ibuprofen | ESI- | 80 | 205.12 → 161.1 (4) | 4000; 13; 320; 25 |
| torasemide | ESI+ | 120 | 349.14 → 263.9/183 (12/36) | 2000; 13; 350; 50 |
| warfarin | ESI+ | 120 | 309.12 → 162.9/250.9 (12/16) | 4500; 13; 350; 30 |
| telmisartan | ESI+ | 230 | 515 → 497/276 (38/40) | 2000; 8; 350; 60 |

# S3: Bioassay results

Caffeine

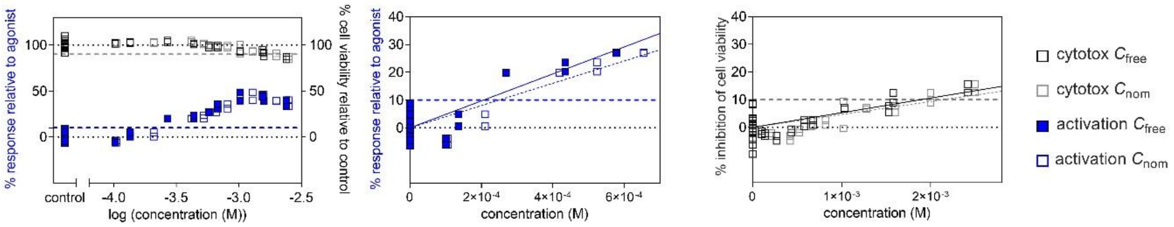


Lamotrigine

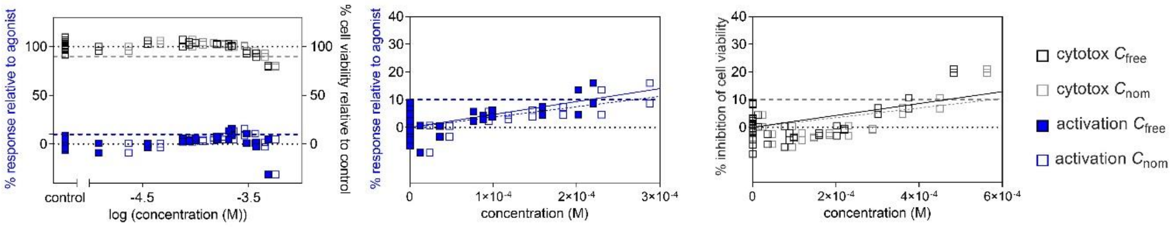


Diclofenac

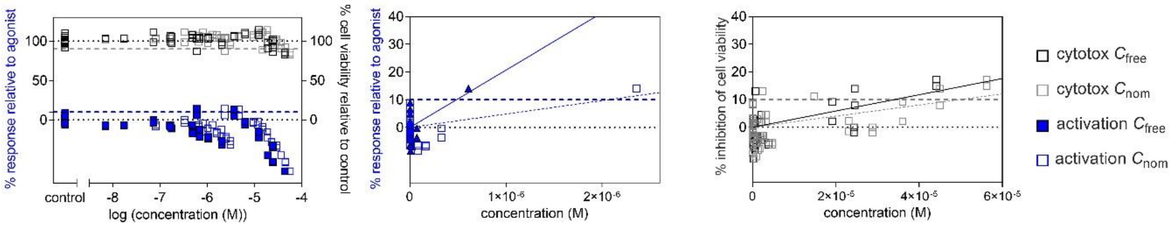


2,4-D

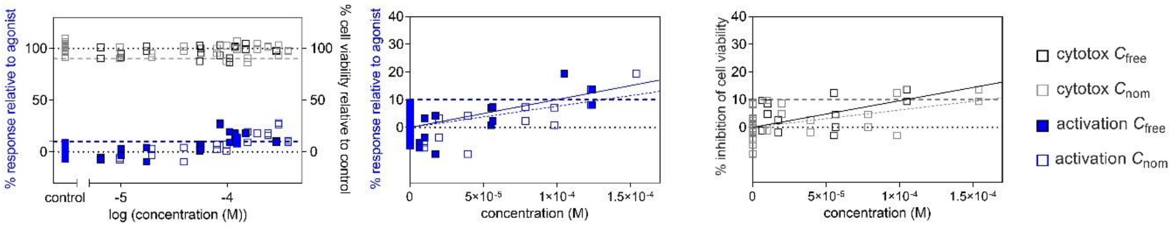


Naproxen

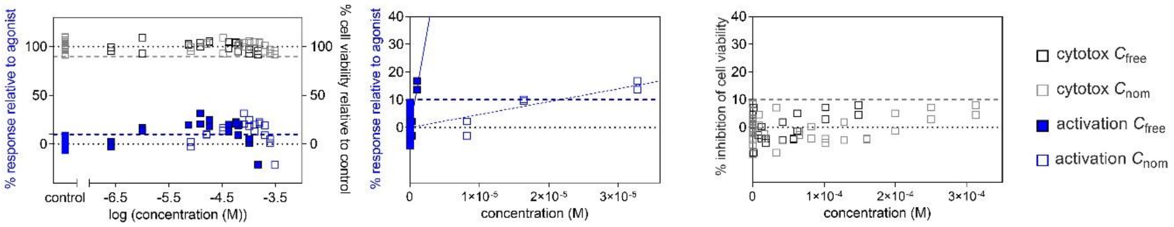

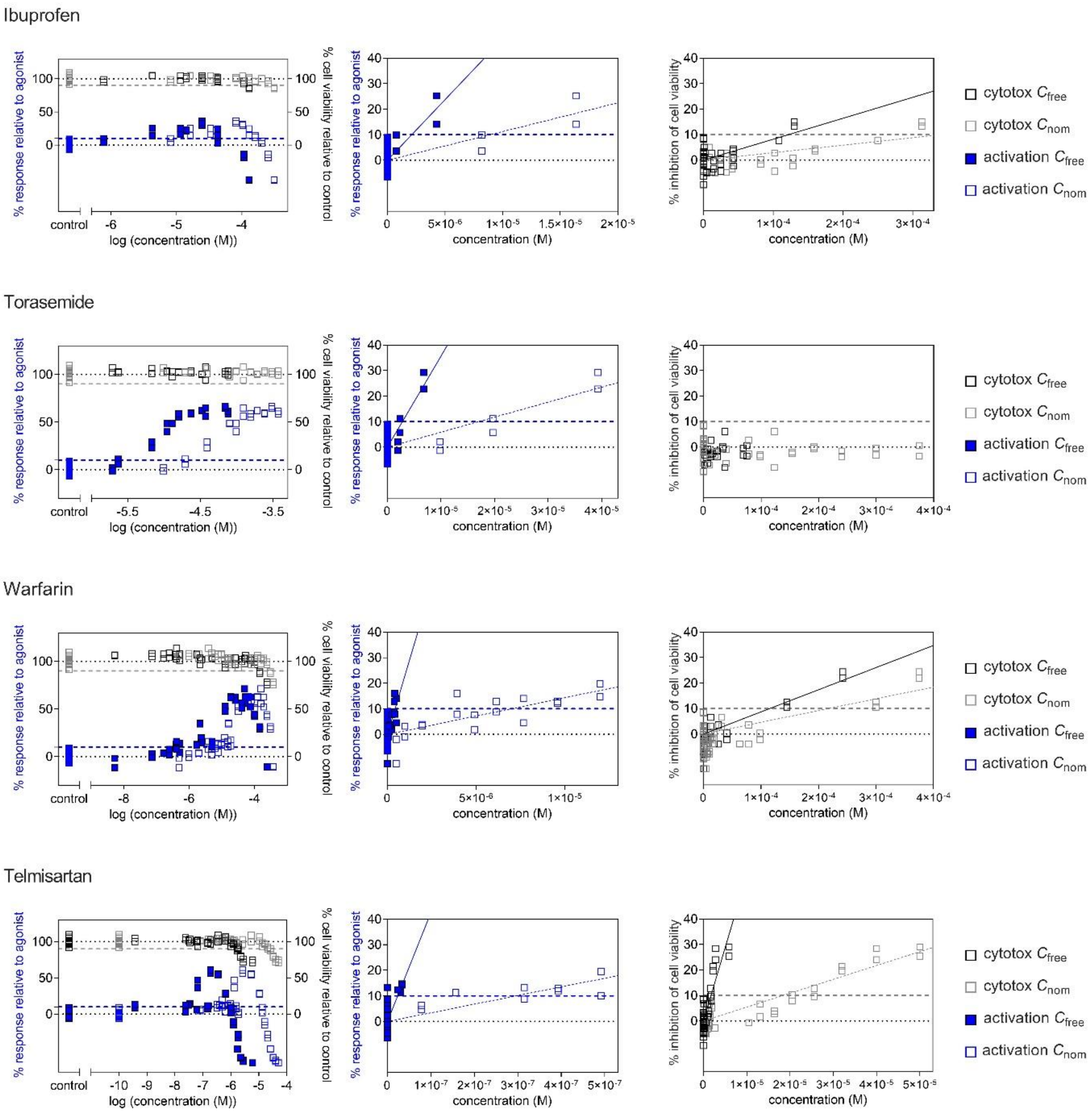


**Figure S1:** Nominal and freely dissolved concentration-response curves of all chemicals obtained from 384-well plates. [Left] Full concentration-response curve for cell viability and induction of the PPARγ. [Middle] Linear range of the concentration-response curve for the induction of the PPARγ at low concentration levels. [Right] Linear range of the concentration-response curve for cytotoxicity

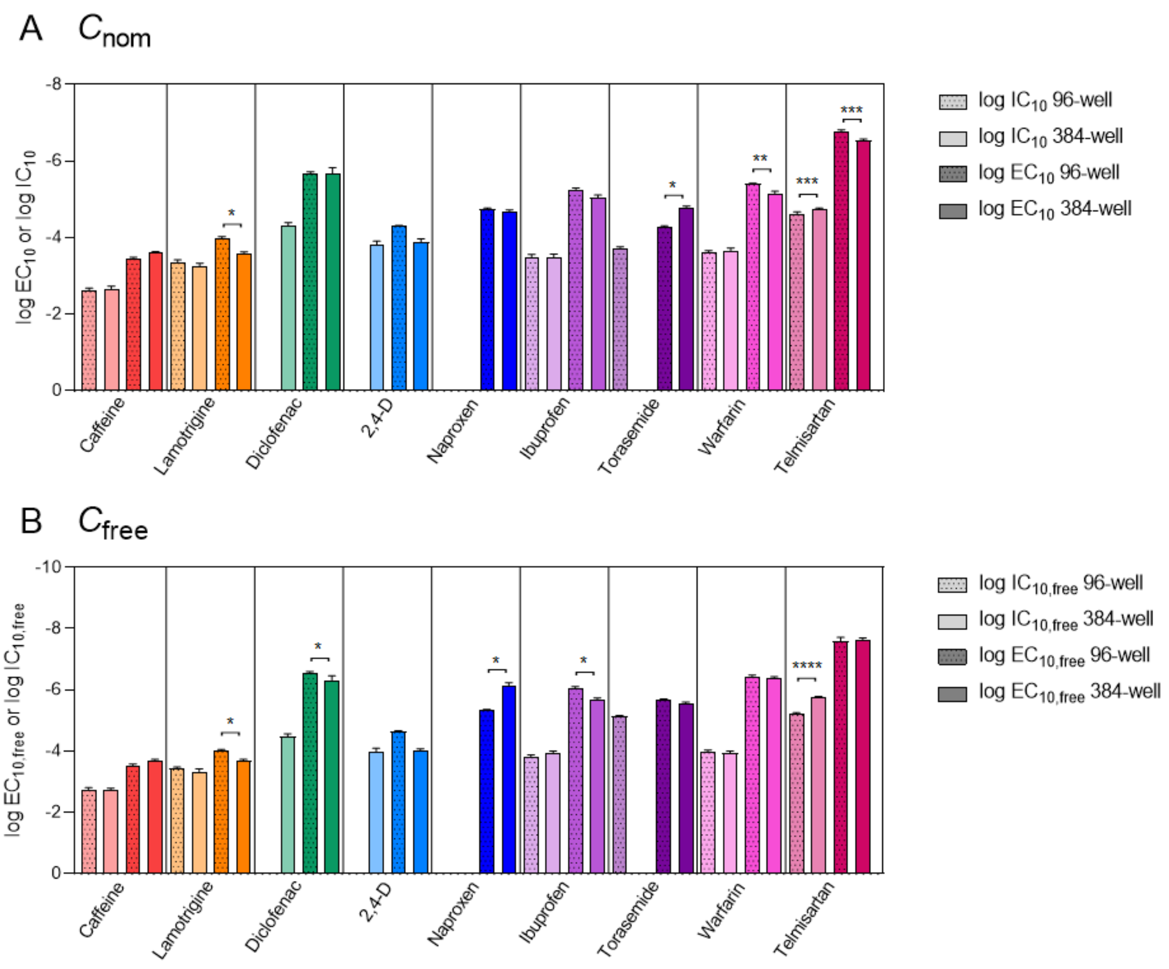


**Figure S2:** Unpaired t-test of log $IC_{10}$ and log $EC_{10}$ of the test chemicals obtained from 96-well plates (Huchthausen et al, 2020) compared to 384-well plates (A). Unpaired t-test of log $IC_{10,free}$ and log $EC_{10,free}$ of the test chemicals obtained from 96-well plates (Huchthausen et al, 2020) compared to 384-well plates (B).

**Table S4:** P-values of unpaired t-test of log $IC_{10}$, $IC_{10,free}$ and log $EC_{10}$ or $EC_{10,free}$ of the test chemicals obtained from 96-well plates (Huchthausen et al, 2020) compared to 384-well plates.

| ***Chemical*** | ***P-value unpaired t-test 96 vs. 384 of $IC_{10}$*** | ***P-value unpaired t-test 96 vs. 384 of $EC_{10}$*** | ***P-value unpaired t-test 96 vs. 384 of $IC_{10,free}$*** | ***P-value unpaired t-test 96 vs. 384 of $EC_{10,free}$*** |
|---|---|---|---|---|
| caffeine | 0.3601 | 0.0839 | 0.7278 | 0.0572 |
| lamotrigine | 0.2814 | 0.0374 | 0.3713 | 0.0492 |
| diclofenac | | 0.9048 | | 0.0380 |
| 2,4-D | | 0.0924 | | 0.0517 |
| naproxen | | 0.3017 | | 0.0405 |
| ibuprofen | 0.7462 | 0.0910 | 0.3761 | 0.0325 |
| torasemide | | 0.0272 | | 0.1509 |
| warfarin | 0.3457 | 0.0016 | 0.3059 | 0.0960 |
| telmisartan | 0.0003 | 0.0004 | 0.0001 | 0.4752 |

## S4: References


Huchthausen, J., Mühlenbrink, M., König, M., Escher, B. I. & Henneberger, L. (2020) Experimental Exposure Assessment of Ionizable Organic Chemicals in In Vitro Cell-Based Bioassays. *Chemical Research in Toxicology*, 33(7), 1845-1854. doi 10.1021/acs.chemrestox.0c00067

Niu, L., Henneberger, L., Huchthausen, J., Krauss, M., Ogefere, A. & Escher, B. I. (2022) pH-Dependent Partitioning of Ionizable Organic Chemicals between the Silicone Polymer Polydimethylsiloxane (PDMS) and Water. *ACS Environmental Au*, 2(3), 253–262. doi: 10.1021/acsenvironau.1c00056

Shedlovsky, T. (1962) The behaviour of carboxylic acids in mixed solvents, in Pesce, B. (ed), *Electrolytes*. New York: Pergamon Press, 146–151.

Yasuda, M. (1959) Dissociation Constants of Some Carboxylic Acids in Mixed Aqueous Solvents. *Bulletin of the Chemical Society of Japan*, 32(5), 429-432. doi: 10.1246/bcsj.32.429
